# Supplementary material for: Use of intravenous immunoglobulin in antiphospholipid antibody positive patients with high risk of miscarriage: a systematic review and meta-analysis
Source: PeerJ. 2024 Oct 31;12:e18419. doi: 10.7717/peerj.18419 (PMC11531756; doi:10.7717/peerj.18419)
Supplement: Supplemental Information 1 [file peerj-12-18419-s001.docx]

| **Section and Topic** | **Item #** | **Checklist item** | **Location where item is reported** |
| --- | --- | --- | --- |
| **TITLE** | | |  |
| Title | 1 | Use of Intravenous Immunoglobulin in Antiphospholipid Antibody Positive Patients with High Risk of Miscarriage: A Systematic Review and Meta-analysis. | 1 |
| **ABSTRACT** | | |  |
| Structured Summary | 2 | The abstract includes background, objectives, data sources, study eligibility criteria, participants, interventions, study appraisal, synthesis methods, results, limitations, conclusions, and implications. | 1,2,3 |
| **INTRODUCTION** | | |  |
| Rationale | 3 | Positivity of aPL in pregnant women is a high-risk factor for miscarriage, and IVIG treatment has emerged as a potential intervention. | 4,5 |
| Objectives | 4 | To evaluate the efficacy of intravenous immunoglobulin (IVIG) in aPL-positive high-risk miscarriages. | 6 |
| **METHODS** | | |  |
| Eligibility criteria | 5 | Included randomized controlled clinical trials assessing the efficacy of IVIG in aPL-positive patients with a high risk of miscarriage. | 7 |
| Information sources | 6 | Databases searched included PubMed, Web of Science, Embase, Scopus, and Medline. | 7 |
| Search strategy | 7 | Detailed search terms and strategy are described. | 8 |
| Selection process | 8 | Two independent reviewers performed study selection. | 9 |
| Data collection process | 9 | Data was extracted by two authors using standardized forms. | 9 |
| Data items | 10 | Extracted data included author, year, country, trial period, sample size, participant characteristics, intervention specifics, and pregnancy outcomes. | 9 |
| Study risk of bias assessment | 11 | Risk of bias was assessed using the Cochrane risk of bias tool. | 9 |
| Effect measures | 12 | Dichotomous data were expressed as odds ratios (ORs) and continuous data as mean differences (MDs), both with 95% confidence intervals (CIs). | 10 |
| Synthesis methods | 13 | Meta-analysis was conducted using Review Manager 5.3, applying fixed or random effects models based on heterogeneity. | 10 |
| Reporting bias assessment | 14 | Publication bias was analyzed for the total effective rate. | 10 |
| **RESULTS** | | |  |
| Study selection | 16 | Flow diagram of study selection is provided (Figure 1). | 11,12 |
| Study characteristics | 17 | Characteristics of included studies are summarized in Table 1. | 13 |
| Risk of bias in studies | 18 | Risk of bias assessment results are presented (Figure 2). | 14 |
| Results of individual studies | 19 | Outcomes of individual studies are presented and discussed. | 15,16 |
| Results of syntheses | 20 | Forest plots for primary and secondary outcomes are provided (Figures 3 and 4). | 17,18 |
| Reporting biases | 21 | Analysis of publication bias is described. | 14 |
| **DISCUSSION** | | |  |
| Discussion | 23a | IVIG intervention shows promise in improving successful pregnancy outcomes and live birth rates in aPL-positive patients with high risk of miscarriage. | 19,20,21,  22,23 |
|  | 23b | Acknowledges the heterogeneity and limitations of the included studies. | 24,25 |
|  | 23c | IVIG appears to be a potentially effective approach for managing aPL-positive pregnant women with a high risk of miscarriage, necessitating further extensive, meticulously designed randomized controlled trials. | 25 |
| **OTHER INFORMATION** | | |  |
| Registration and protocol | 24 | The protocol was registered in the PROSPERO database (registration number CRD42023447838). | 4 |
| Support | 25 | **Funding:** CAMS Innovation Fund for Medical Sciences, Science & Technology Department of Sichuan Province, and Scientific Research Project of Sichuan Medical Association. | 26 |
| Competing interests | 26 | The authors declare no conflicts of interest related to this systematic review. | 25 |
| Availability of data, code and other materials | 27 | **Availability:** All data generated or analyzed during this review are included in this article. | 26 |

*From:*  Page MJ, McKenzie JE, Bossuyt PM, Boutron I, Hoffmann TC, Mulrow CD, et al. The PRISMA 2020 statement: an updated guideline for reporting systematic reviews. BMJ 2021;372:n71. doi: 10.1136/bmj.n71
